# Supplementary material for: Reflection of near-infrared light confers thermal protection in birds
Source: Nat Commun. 2018 Sep 6;9:3610. doi: 10.1038/s41467-018-05898-8 (PMC6127310; doi:10.1038/s41467-018-05898-8)
Supplement: Supplementary file 1 — Supplementary Information [file 41467_2018_5898_MOESM1_ESM.pdf]

Supplementary Information

Reflection of near-infrared light confers thermal protection in birds

Medina et al.

Supplementary Tables

Supplementary Table 1. Eigenvectors and eigenvalues of the first two principal components (PC) from a Principal Components Analysis of 5 climatic variables for each individual (N = 616). Values are averages over the 26 years from 1990 - 2015 inclusive.

VARIABLE	PC1	PC2
Number of days >35°C per year	0.42	0.44
Average maximum summer temperature	0.48	0.40
Average summer solar irradiation	-0.22	0.66
Average winter solar irradiation	0.58	-0.04
Average yearly vapour pressure	0.45	-0.46
**Eigenvalue**	2.8	1.76
**Proportion of variance**	0.56	0.35
**Cumulative proportion**	0.56	0.91

Supplementary Table 2. Results of full PGLS models predicting absolute NIR reflectivity and including UV-visible reflectivity as a covariate across 90 species. Best models are shown for each body patch. 95% confidence intervals across 1000 trees are shown for each predictor. ER refers to the average evidence ratio between the best model (shown) and a model with only visible reflectivity across trees. T% represents the percentage of trees in which the model reported was the best.

Region	Predictor	Slope	t-value	P-value	%T	ER
Mantle	Mass (log g)	-3.11 – -2.43	-3.44 – -2.60	0.0001 – 0.008	99.7	> 1000
	Environmental PC2	2.97 – 3.69	3.16 – 3.77	0.001 – 0.002		
	UV-VIS	0.80 – 0.89	23.98 – 53.82	< 0.0001		
	mass*PC2	-1.35 – -1.03	-2.89 – -2.33	0.004 – 0.02		
Crown	Mass (log g)	-4.86 – -2.82	-4.10 – -2.57	0.001 – 0.006	82.3	> 1000
	Environmental PC2	3.27 – 5.28	3.21 – 4.91	< 0.001		
	UV-VIS	0.87 – 0.95	19.96 – 28.57	< 0.0001		
	mass*PC2	-1.94 – -1.03	-4.25 – -2.38	0.001 – 0.01		
Breast	Mass (log g)	-5.09 – -3.86	-5.12 – -3.88	< 0.001	86.3	> 1000
	Environmental PC1	1.62 – 2.54	2.04 – 4.29	0.001 – 0.03		
	UV-VIS	0.81 – 0.87	23.63 – 29.06	< 0.0001		
	mass*PC1	-1.23 – -0.85	-5.02 – -2.23	0.001 – 0.02		
Belly	Mass (log g)	-6.17 – -4.44	-6.21 – -3.80	< 0.001	84.2	> 1000
	Environmental PC1	0.89 – 2.32	1.06 – 4.48	0.001 – 0.13		
	UV-VIS	0.81 – 0.88	27.12 – 38.67	< 0.0001		
	mass*PC1	-1.13 – -0.53	-5.17 – -1.80	0.001 – 0.04		

Supplementary Table 3. Best models predicting relative NIR (phylogenetic residuals from correlation with UV-visible reflectivity) and UV-visible reflectivity for four body patches across 90 species. 95% confidence intervals across 1000 trees are shown for each predictor. ER refers to the average evidence ratio between the best model (shown) and the null model across trees. T% represents the percentage of trees in which the model reported was the best.

	**Body region**	**Predictor**	**Slope**	**t-value**	**P-value**	**ER**	**T%**
**Relative NIR**	Mantle	Mass (log g)	-2.06 – 1.52	-2.55 – -1.83	0.003 – 0.04	119	89
		Environmental PC2	2.89 – 3.24	2.77 – 3.27	0.001 – 0.006		
		Mass * PC2	-1.18 – -0.98	-2.49 – -1.97	0.013 – 0.05		
	Crown	Mass (log g)	-3.12 – -1.41	-3.03 – -1.52	0.002 – 0.112	865	90
		Environmental PC2	3.33 – 4.59	2.76 – 4.52	0.001 – 0.005		
		Mass * PC2	-2.06 – -0.96	-4.71 – -1.69	0.001 – 0.08		
	Breast	Mass (log g)	-2.94 – -1.94	-3.24 – -2.01	0.001 – 0.04	7.6	81
		Environmental PC1	1.60 – 2.52	2.00 – 4.27	0.001 – 0.03		
		Mass * PC1	-1.36 – -0.89	-5.59 – -2.29	0.001 – 0.01		
	Belly	Mass (log g)	-4.40 – -2.24	-5.26 – -2.08	0.001 – 0.03	16	82
		Environmental PC1	0.95– 2.36	1.55 – 5.24	0.001 – 0.06		
		Mass * PC1	-1.29 – -0.67	-6.10 – -2.22	0.001 – 0.014		
**UV-Visible**	Mantle	Environmental PC2	2.47 – 2.73	2.17 – 2.44	0.016 – 0.031	4.65	53
	Crown		No significant predictors			–	100
	Breast		No significant predictors			–	100
	Belly	Mass (log g)	-9.64 – -7.79	-2.57 – -2.20	0.01 – 0.03	5.96	86

Supplementary Table 4. Results of species level analyses (PGLS) on the relationship between habitat category and relative NIR and UV-visible reflectivity. Significant values are highlighted in bold. Arid species have higher NIR of the mantle and crown than species in other habitat categories.

		RELATIVE REFLECTIVITY			VISIBLE REFLECTIVITY		
	**Predictor**	**Slope**	**T-value**	**P-value**	**Slope**	**T-value**	**P-value**
MANTLE	Arid vs. Forest	-3.25 – -2.94	-2.05 – -1.85	**0.04 – 0.06**	-9.60 – -8.69	-2.12 – -1.88	**0.04 – 0.06**
	Arid vs. Generalist	-3.07 – -2.84	-2.62 – -2.43	**0.01 – 0.02**	-4.46 – -3.42	-1.30 – -1.05	0.19 – 0.29
	Arid vs. Shore	-6.12 – -5.54	-3.06 – -2.53	**0.002 – 0.012**	-3.98 – -1.51	-0.55 – -0.22	0.58 –0.82
	Arid vs. Water	-5.34 – -4.87	-2.61 – -2.42	**0.01 – 0.02**	6.80 – 10.77	1.06 – 1.57	0.11 – 0.29
	Mass (log g)	-1.98 – -1.15	-2.44 – -1.53	0.08 – 0.11	-2.87 – -2.35	-1.13 – -0.92	0.26 – 0.35
CROWN	Arid vs. Forest	-4.65 – -2.13	-2.54 – -1.41	**0.001 – 0.13**	-3.15 – -0.97	-0.80 – -0.25	0.40 – 0.77
	Arid vs. Generalist	-4.53 – -2.81	-3.55 – -2.81	**0.0002 – 0.004**	-4.12 – -1.48	-1.46 – -0.57	0.12 – 0.52
	Arid vs. Shore	-7.82 – -6.60	-3.56 – -1.73	**0.0002 – 0.08**	-2.77 – 1.78	-0.32 – 0.19	0.77 – 0.99
	Arid vs. Water	-5.87 – -3.06	-2.61 – -0.86	**0.009 – 0.38**	13.04 – 19.78	1.53 – 2.61	0.004 – 0.11
	Mass (log g)	-2.64 – -0.06	-2.59 – -0.29	0.06 – 0.72	-3.21 – -1.76	-1.13 – -0.61	0.24 – 0.51
BREAST	Arid vs. Forest	-0.30 – 0.14	-0.17 – 0.08	0.87 – 0.99	9.65 – -7.93	-1.67 – -1.39	0.08 – 0.15
	Arid vs. Generalist	-2.59 – -2.09	-1.98 – -1.69	0.05 – 0.9	-4.23 – -2.42	-0.99 – -0.62	0.32 – 0.53
	Arid vs. Shore	-5.87 – -5.28	-2.46 – -1.98	**0.01 – 0.05**	2.68 – 5.82	0.28 – 0.72	0.47 – 0.77
	Arid vs. Water	-2.35 – -1.45	-0.98– -0.55	0.32 – 0.57	2.77 – 7.72	0.34 – 0.84	0.39 – 0.73
	Mass (log g)	-1.89 – -1.64	-2.02 – -1.73	**0.04 – 0.08**	-7.36 – -5.59	-2.24 – -1.81	0.025 – 0.07
BELLY	Arid vs. Forest	0.45 – 1.55	0.25 – 1.01	0.31 – 0.79	-10.11 – -8.56	-1.54 – -1.19	0.14 – 0.22
	Arid vs. Generalist	-2.03 – 0.63	-1.61 – -0.62	0.09 – 0.47	-2.02 – -0.97	-0.38 – -0.20	0.70 – 0.84
	Arid vs. Shore	-4.30 – -3.36	-1.44 – -0.99	0.15 – 0.32	10.81 – 14.42	1.07 – 1.71	0.09 – 0.28
	Arid vs. Water	-3.21 – -1.89	-1.12 – -0.62	0.24 – 0.52	3.91 – 5.95	0.43 – 0.61	0.53 – 0.66
	Mass (log g)	-2.22 – -1.71	-2.04 – -1.62	0.04 – 0.10	-10.27 – -7.60	-2.75– -2.33	0.006 – 0.65

Supplementary Table 5. Results of full PGLS models predicting relative and absolute NIR reflectivity when all body patches are combined. 95% confidence intervals across 1000 trees are shown for each predictor in the best model. ER refers to the average evidence ratio between the best model (shown) and the null model across trees. T% represents the percentage of trees in which the model reported was the best.

	**Body region**	**Predictor**	**Slope**	**t-value**	**P-value**	**ER**	**T%**
**Relative NIR**	Combined	Mass (log g)	-3.38 – -1.41	-4.03 – -1.79	0.0002 – 0.07	>1000	89%
		Environmental PC1	0.95 – 1.75	1.76 – 3.58	0.0001 – 0.04		
		Environmental PC2	1.87 – 2.74	2.24 – 3.35	0.001 – 0.02		
		Mass * PC1	-0.87 – -0.38	-3.92 – -1.72	0.0001 – 0.06		
		Mass * PC2	-1.36 – -0.74	-3.57 – -1.93	0.0001 – 0.04		
**NIR absolute**	Combined	Mass (log g)	-4.47 – -3.07	-5.28 – -3.68	0.0001 – 0.0003	>1000	86%
		Environmental PC1	7.23– 1.81	1.25 – 4.29	0.0001 – 0.10		
		Environmental PC2	1.81 – 2.94	2.37 – 3.69	0.0001 – 0.02		
		Mass * PC1	-0.87 – -0.34	-4.52 – -1.61	0.0001 – 0.07		
		Mass * PC2	-1.34 – -0.63	-3.57 – -1.86	0.0001 – 0.05		
		UV-visible reflectivity	0.81- 0.88	25.52 – 31.84	< 0.0001		

Supplementary Table 6. Best models predicting relative NIR reflectivity for four body patches across 90 species, for each sex separately. 95% confidence intervals across 1000 trees are shown for each predictor. ER refers to the average evidence ratio between the best model (shown) and the null model across trees. T% represents the percentage of trees in which the model reported was the best. F = female, M = male.

Sex/Region	Predictor	Slope	t-value	P-value	%T	ER
F Crown	PC2	2.63 – 2.87	2.36 – 2.69	0.008 – 0.02	100	25.7
	mass	-1.77 – -1.74	-1.95 – -1.73	0.05 – 0.08		
	PC2 * mass	0.98 – -0.84	-1.93 – -1.60	0.05 – 0.11		
M Crown	PC2	3.89 – 5.031	2.80 – 3.70	0.001 – 0.005	89	69
	mass	-2.86 – -1.47	-2.13 – -1.28	0.03 – 0.188		
	PC2 * mass	-1.87 – -1.34	-3.07 – -1.88	0.006 – 0.04		
F Mantle	PC2	1.22 – 1.36	1.22 – 1.38	0.17 – 0.22	100	38.4
	mass	-2.24 – -2.08	-3.01 – -2.78	0.003 – 0.006		
M Mantle	PC2	4.39 – 4.92	3.45 – 3.90	0.001 – 0.007	100	503
	mass	-1.97 – -1.55	-1.91 – -1.55	0.05 – 0.11		
	PC2 * mass	-1.70 – -1.49	-2.93 – -2.52	0.003 – 0.01		
F Breast	mass	-3.15 – -1.84	-2.81 – -1.72	0.01 – 0.07	100	1.7
M Breast	PC1	2.36 – 3.79	2.67 – 7.02	0.001 – 0.005	95.1	8.29
	mass	-1.18 – -0.17	-1.63 – -0.17	0.07– 0.81		
	PC1 * mass	-1.83 – -1.26	-8.01 – -3.07	0.001 – 0.002		
F Belly	PC1	1.67 – 2.18	2.54 – 3.12	0.002 – 0.012	100	13.36
	mass	-2.43 – -1.86	-2.33 – -1.81	0.02 – 0.07		
	PC1 * mass	-1.01 – -0.82	-3.45 – -2.57	0.001 –0.008		
M Belly	PC1	1.60 – 4.03	2.09 – 7.79	0.001 – 0.006	98	44
	mass	-3.37 – -1.66	-3.16 – -1.47	0.001 – 0.10		
	PC1 * mass	-1.99 – -1.02	-9.08 – -3.05	< 0.0001		

Supplementary Table 7. Results of full PGLS models predicting total reflectivity (UV + Visible + NIR) by body patch. 95% confidence intervals across 1000 trees are shown for each predictor in the best model. ER refers to the average evidence ratio between the best model (shown) and the null model across trees. T% represents the percentage of trees in which the model reported was the best.

	Body region	Predictor	Slope	t-value	P-value	ER	T%
**Total reflectivity**	Mantle	Environmental PC2	2.88 – 3.17	2.63 – 2.97	0.003 – 0.009	14.52	100
	Crown	Environmental PC2	1.30 – 2.00	1.44 – 2.06	0.03 – 0.15	1.63	85.6
	Belly	Mass (log g)	-11.20 – -9.34	-3.20 – -2.83	0.001 – 0.005	29.92	89
	Breast	Mass (log g)	-8.24 – -4.07	-5.50 – -2.31	< 0.0001 – 0.02	16	82

Supplementary Table 8. List of variables and parameters used in endotherm models for Australia.

	Units	Value	Source
**Environmental variables**			
Air temperature	°C	14 to 50	This study
Wind velocity	m/s	0.15 to 3.7	This study
Humidity	%	3.6 to 91	This study
Solar radiation	W/m^2	0 to 1103	This study
Zenith angle of sun	°from overhead	4.5 to 90	This study
Solar absorptivity of substrate	%	70	This study
**Biophysical variables**			
Mass	kg	0.01, 0.1 and 1	This study
Feather depth	mm	1.4 to 20	Estimated
Feather length	mm	13 to 52	Estimated
Dorsal reflectivity	%	10 or 40	This study
Ventral reflectivity	%	40	This study
Percent of skin acting as free water surface	%	0.05	Kearney et al. 2016
Percent of sweat increment	%	0.1	Kearney et al. 2016
Body shape	length/width	3	Estimated

Supplementary Figures

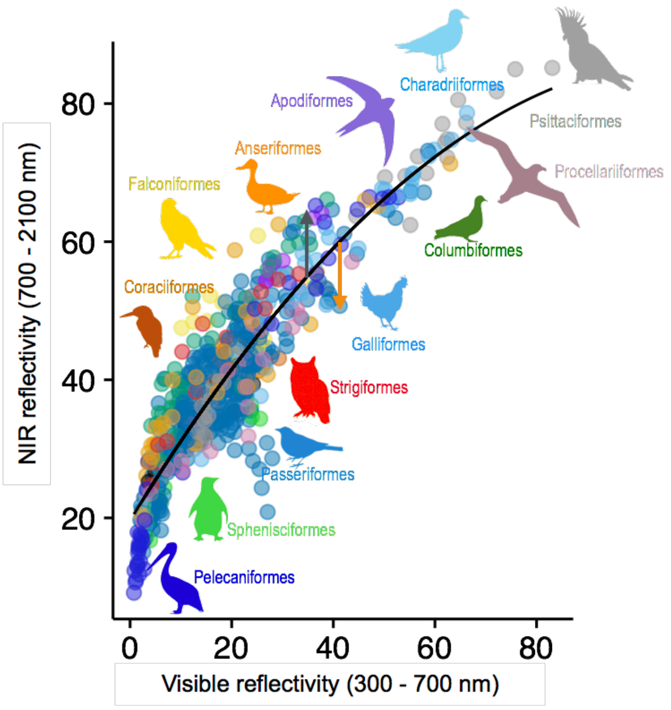

Supplementary Figure 1. Association between NIR and UV-visible reflectivity coloured by avian order. Quadratic fit shown, red arrows represent how residuals were calculated to derive ‘relative NIR’. Silhouettes obtained from www.pixabay.com or Wikimedia commons.

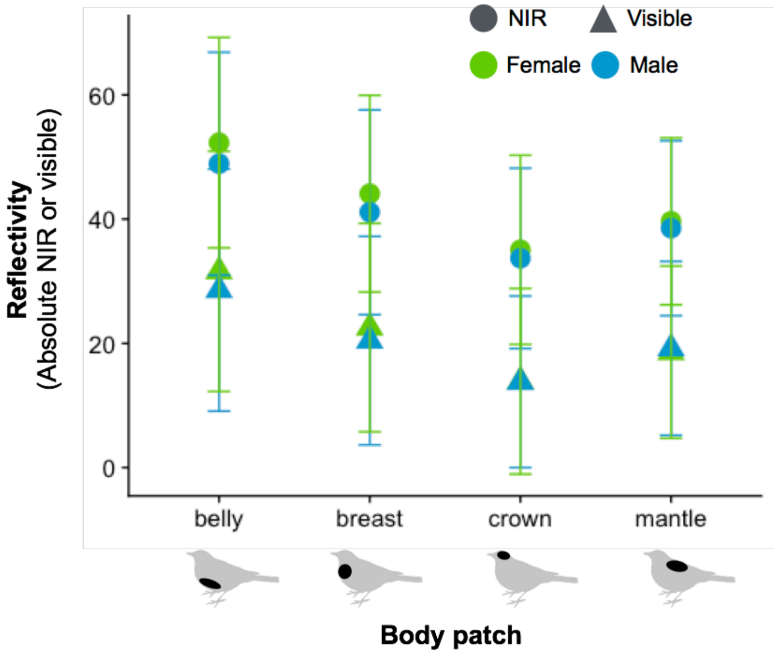

Supplementary Figure 2. Values of NIR and UV-visible reflectivity for males and females. In NIR reflectivity there are significant differences for the belly (GLMM , β=3.28, p< 0.001) and breast (GLMM , β =2.71, p < 0.001) but not for the crown (GLMM , β =-0.815, p=0.06) and mantle (GLMM , β =-0.72, p=0.22). There are no differences for visible reflectivity in the mantle (β =0.76, p=0.19) and crown (β =0.43, p=0.24), but there are significant differences in the breast (GLMM, β =-1.64, p<0.001) and in the belly (GLMM , β =-2.87, p<0.001).

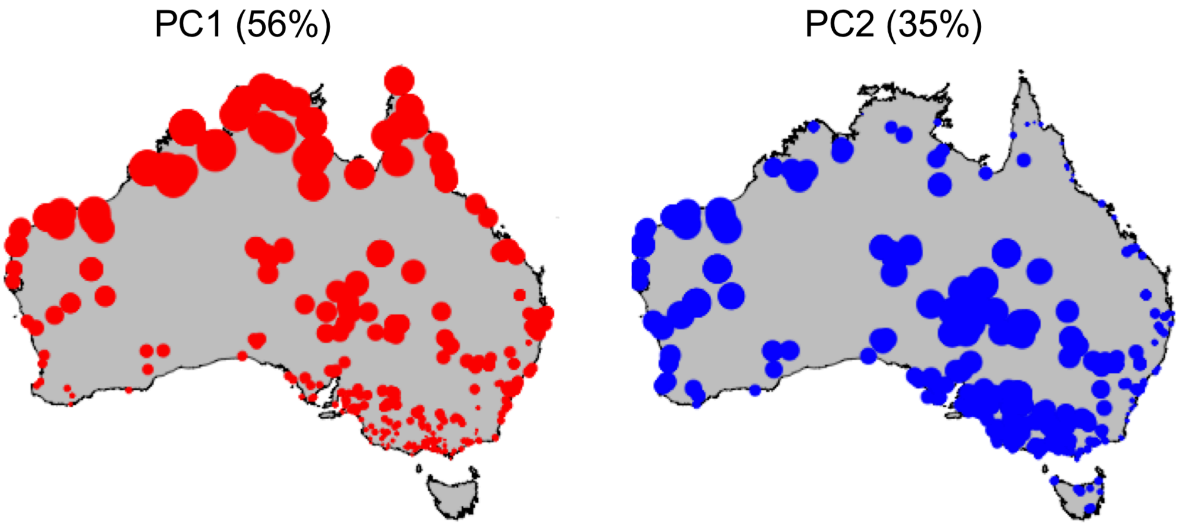

Supplementary Figure 3. Loadings for each environmental Principal Component (PC) at each specimen locality. The size of circle is proportional to the value for each PC. Higher values of PC1 are found in tropical areas and higher values of PC2 are found in arid zones.

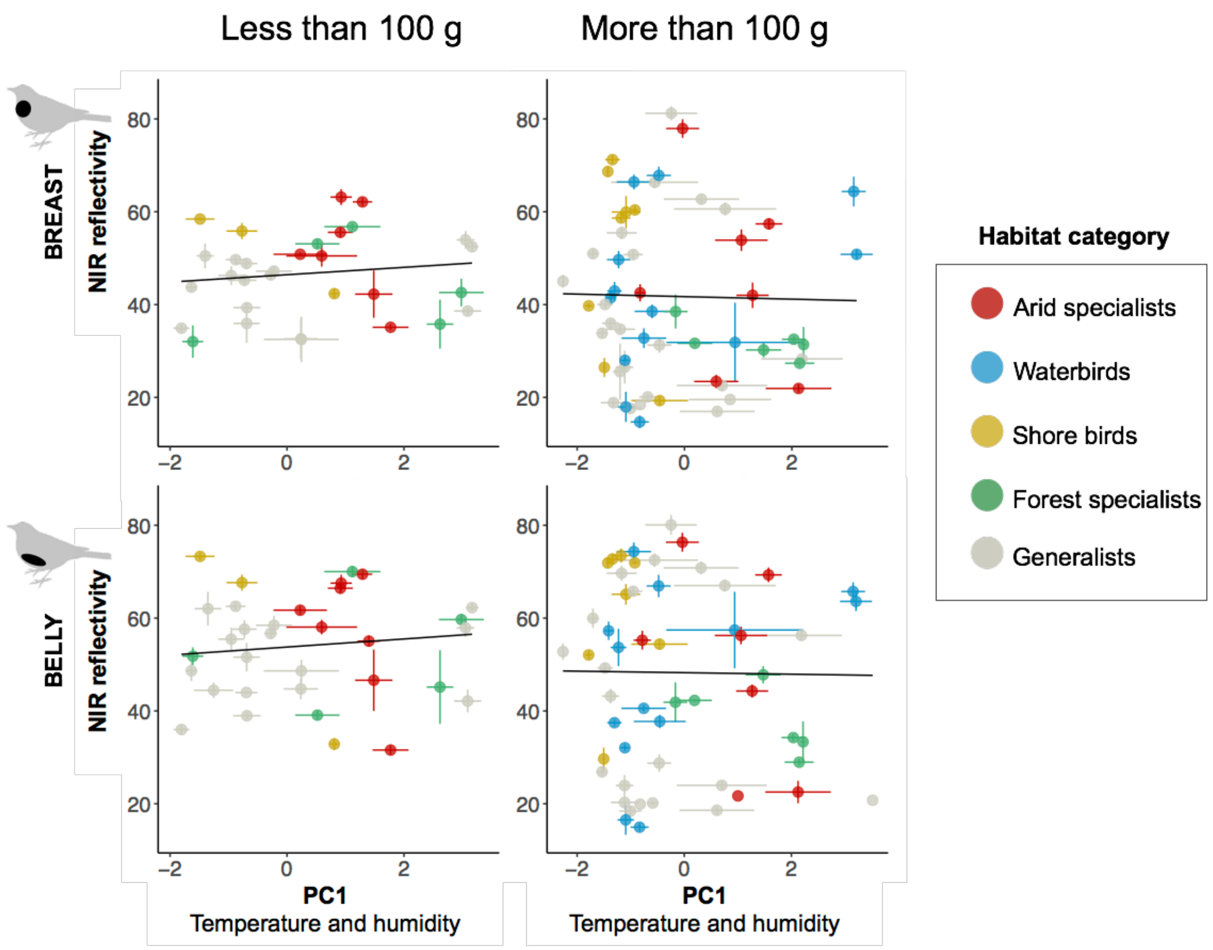

Supplementary Figure 4. Relationship between environmental variables (PC1) and NIR reflectivity in two ventral body patches. Each point represents the average value per species and the standard error in both axes, colour symbolises the species habitat. Trend lines were predicted using a phylogenetically controlled model that included environmental PCs, visible reflectivity, and a random phylogenetic tree.

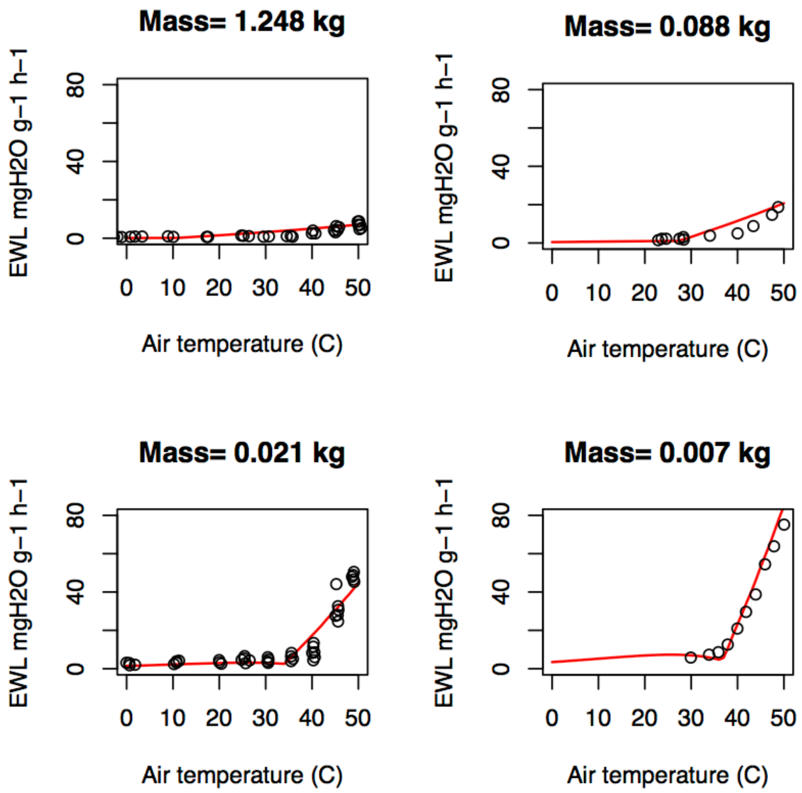

Supplementary Figure 5. Model validation results using published physiological data from Wolf and Walsberg[2]. Points represent observed values of water loss obtained in the metabolic chamber, red lines represent the values of EWL predicted by the endotherm model used in this study.

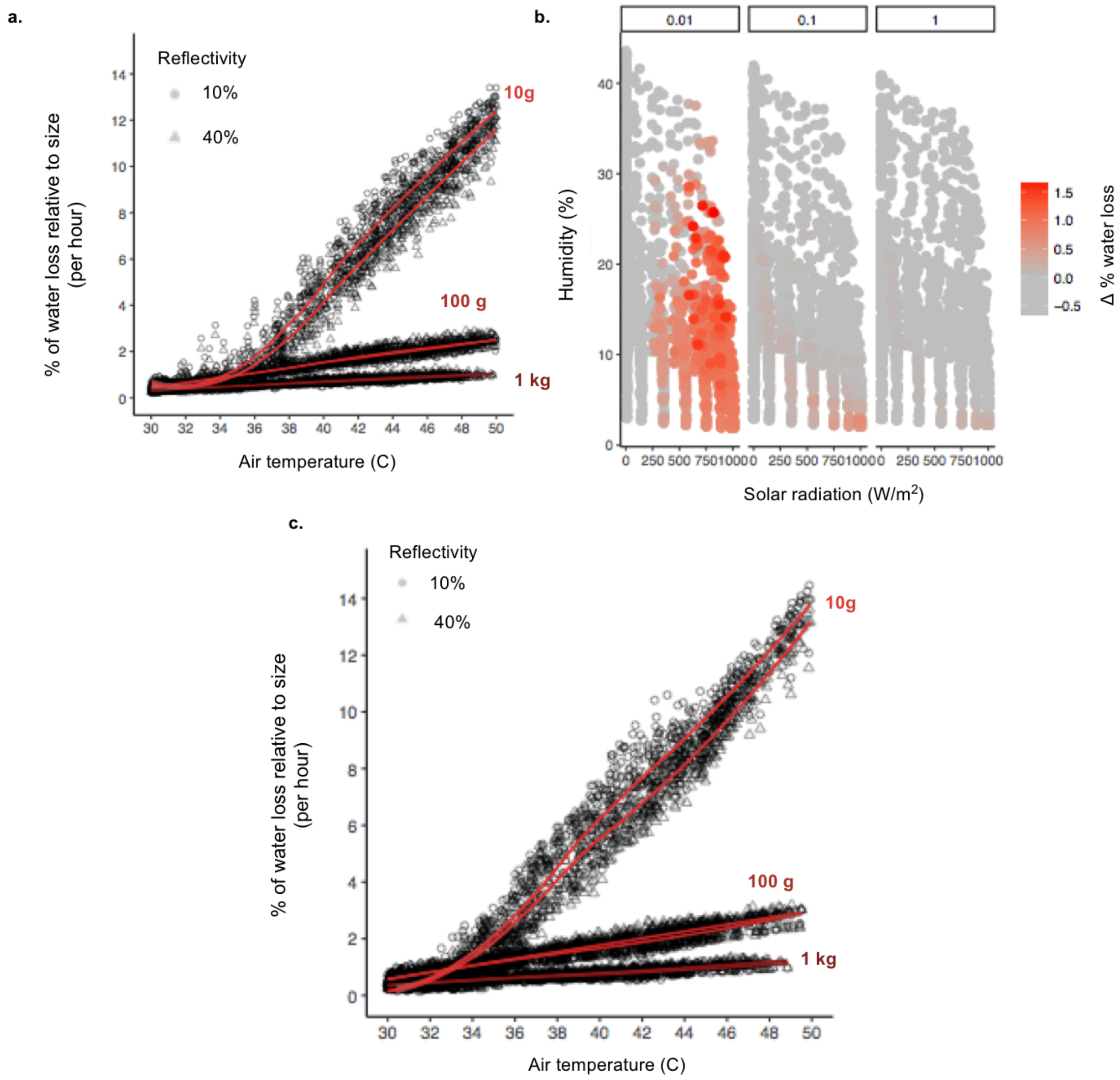

Supplementary Figure 6. Biophysical model results in Arizona, USA. A. Relationship between air temperature and water loss according to body mass and total reflectivity. B. Climate space plot showing the difference in the percentage of water loss when reflectivity was 10% vs. 40%. Higher values (warmer colours) indicate a higher percentage of water saved by having higher total reflectivity values. Gray values (closer to zero) indicate no difference in water loss between lower and higher reflectivity. The highest difference in water loss between high and low reflectivity values was 1.69% body weight per hour for 10 g, 0.68% for 100 g and 0.25% for 1 kg birds, similar values to those for Australia. C. Relationship between air temperature and water loss according to body mass and total reflectivity in Australia (Simpson Desert).

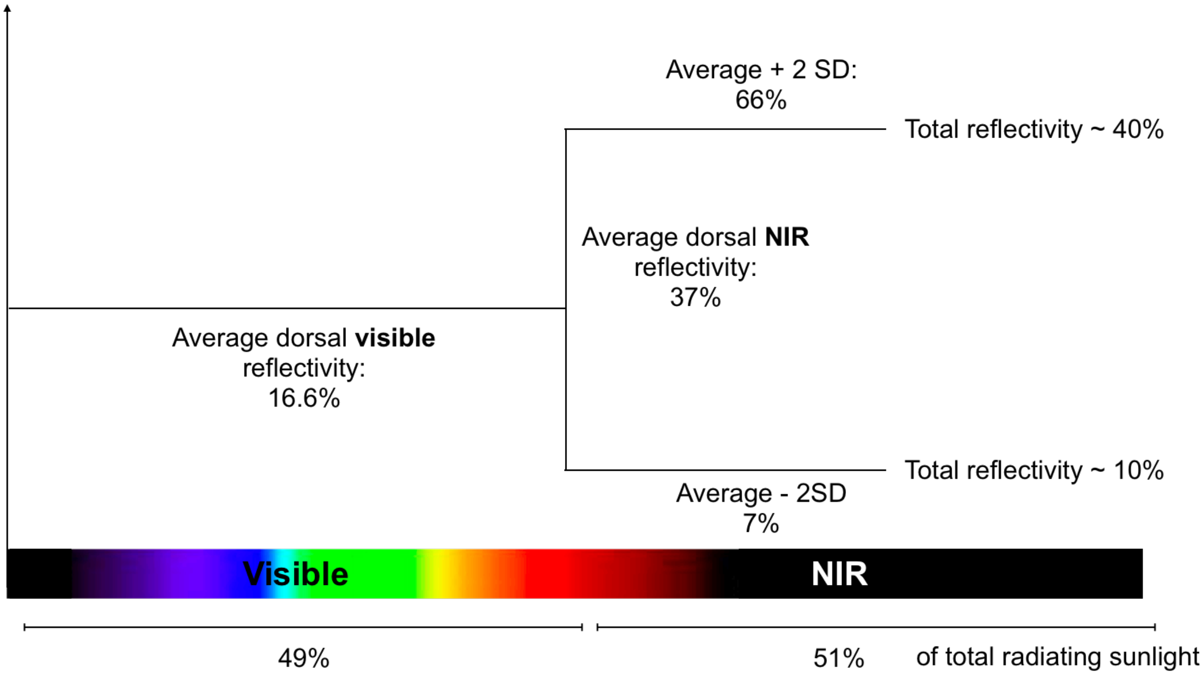

Supplementary Figure 7. Schematic view of reflectivity values chosen for endotherm models. Average value of UV-visible reflectivity was weighted by the percentage of total radiating sunlight in that region (49%), and the values for NIR were also weighted accordingly (51%). Values of 40% and 10% represent the upper and lower 95% interval of the variation in NIR in our dataset. Ventral reflectivity was kept constant at a value of 40%, which is the average value for combined visible and NIR ventral reflectivity in our dataset.

Supplementary References

1 Shawkey, M. D. & D'Alba, L. Interactions between colour-producing mechanisms and their effects on the integumentary colour palette. *Phil. Trans. R. Soc. B* **372**, 20160536, doi:10.1098/rstb.2016.0536 (2017).

2 Wolf, B. O. & Walsberg, G. E. The Role of the Plumage in Heat Transfer Processes of Birds. *Am. Zool.* **40**, 575-584, doi:10.1093/icb/40.4.575 (2000).
