# Supplementary material for: Reflection of near-infrared light confers thermal protection in birds
Source: Nat Commun. 2018 Sep 6;9:3610. doi: 10.1038/s41467-018-05898-8 (PMC6127310; doi:10.1038/s41467-018-05898-8)
Supplement: Supplementary file 3 — Description of Additional Supplementary Files [file 41467_2018_5898_MOESM3_ESM.pdf]

Description of Additional Supplementary Files

File Name: **Supplementary Data 1**
Description: **Species used in the study**. Information on species name, sample size and habitat for the 90 species analysed in the study.
